# Personalized auditory cues improve gait in patients with early Parkinson's disease

**DOI:** 10.3389/fneur.2025.1561880

**Published:** 2025-06-18

**Authors:** Xinjin Li, Shiya Wang, Kai Wang, Wenjie Wang, Houzhen Tuo, Yixin Long, Xiaohui Tan, Wei Sun

**Affiliations:** ^1^Institute of Software Research, Chinese Academy of Sciences, Beijing, China; ^2^School of Computer Science and Technology, University of Chinese Academy of Sciences, Beijing, China; ^3^Department of Neurology, Beijing Friendship Hospital, Capital Medical University, Beijing, China; ^4^Information Engineering College, Capital Normal University, Beijing, China; ^5^School of Medical Humanities, Capital Medical University, Beijing, China

**Keywords:** gait disorder, rhythmic entrainment, personalized music, gait sensing, Parkinson's disease

## Abstract

**Introduction:**

Parkinson's disease (PD) patients experience a wide variety of gait and posture problems that significantly impair their functional mobility and quality of life. Auditory cue-based training has been shown to improve gait performance in PD patients. However, most of the current methods target gains in bilateral spatiotemporal variables, whereas in the early-stages of PD, symptoms are usually unilateral.

**Methods:**

To address the effects of unilateral onset and heterogeneity of early-stage PD on patients' gait performance, we propose a personalized training method based on auditory cues to reduce gait asymmetry between patients' right and left feet. The method targets patients' gait performance through personalized music (auditory cues) and dynamically adjusts the music based on real-time gait data to ensure synchronization with the patient's walking rhythm. Specifically, gait data are acquired in real time via Inertial Measurement Units (IMUs) attached to the ankles of the patient's right and left feet, which are used to calculate the gait cycles of the patient's right and left feet. Personalized music is then generated based on the patient's gait cycle. During the training process, the music is dynamically updated by continuously assessing the synchronization between the patient's gait cycle and the music beats.

**Results:**

Fifteen early-stage PD patients(H&Y ≤ 2.5) were initially recruited to compare and analyze the effects of training with and without auditory cues. Gait symmetry improved in all patients who received auditory cues (t = 4.9166, *p* = 0.0002), with a maximum improvement of 17.685%, and gait variables also showed significant enhancement. Eleven early-stage patients were then recruited for a 7-day intervention, with a mean improvement in gait symmetry of 11.803% (*t* = 4.391, *p* = 0.001). There were significant improvements in left-foot velocity (*t* = 4.613, *p* = 0.001), right-foot velocity (t = 6.250, *p* = 0.0001), and right-foot stride length (*t* = 4.004, *p* = 0.0025), and the average improvement rate of gait variables reached 37.947%. This indicates that the personalized training method proposed in this paper for the unilateral onset characteristics of early-stage PD is effective. It not only enhances the symmetry of walking in patients with early-stage PD, but also improves motor performance.

**Discussion:**

The proposed method can serve as a complementary approach to pharmacological treatment in the rehabilitation of PD patients, demonstrating its effectiveness in clinical application.

## 1 Introduction

Parkinson's disease (PD) is a progressive neurodegenerative disorder, with its incidence rate having increased by 2.5 times over the past three decades (1, 2), affecting approximately 0.3% of the global population (3). PD is caused by the progressive loss of dopaminergic neurons in the substantia nigra, resulting in basal ganglia dysfunction (4). PD patients often experience a variety of gait and posture problems, which significantly impair their functional mobility and quality of life (5–9). These abnormal gait and posture patterns may result from dysfunctions in the motor control system regulated by the cortical-striatal loop (10). Fortunately, research has found that PD patients can utilize external cues to provide spatial information to guide movement, allowing them to bypass their defective basal ganglia while walking (11), thereby regulating gait. Meanwhile, clinical guidelines, particularly in physiotherapy, emphasize the critical role of training in managing both motor and non-motor symptoms (12–14). There is growing recognition of the importance of training in the management of the disease (15). However, due to the strong heterogeneity of PD, personalized training programs are of great importance for the management and treatment of PD (16).

The auditory and motor systems are richly interconnected at various cortical, subcortical, and spinal (17). Auditory cues provide external rhythmic stimulation, bypassing the basal ganglia, and synchronize gait with external rhythms in both time and space through the auditory-motor neural network. In this process, central pattern generators can produce walking rhythms synchronized with musical rhythms, controlling muscle tone, which aids in improving the abnormal gait patterns of patients with PD (18). The results of functional magnetic resonance imaging also indicate that the cerebral cortex is activated during beat perception of musical rhythms, which in turn leads to increased connectivity between motor and auditory areas of the cerebral cortex (19). Braunlich et al. (20) found that auditory stimulation can enhance velocity, step frequency, and stride length. The study by Thaut et al. (21) indicates that training with a metronome can significantly improve patients' stride length, double stance phase, step frequency, and velocity. Wu et al. (22) discovered that music therapy is an effective method for treating gait disorders caused by PD, with mechanisms of action including rhythm induction, neural coherence stimulation, and acceleration of motor learning. Cancela et al. (23) found that symptoms in PD patients can be improved through rehabilitation exercises based on auditory cues. Gondo et al. (24) observed that PD patients walking with music gradually increased in acceleration, velocity, stride length, and experienced a reduction in medio-lateral amplitude during walking. Sweeney et al. (25) found that auditory cues can effectively help PD patients recover from freezing of gait. Although these studies have validated the effectiveness of auditory cues in improving gait disorders in PD, these methods primarily target the improvement of bilateral spatiotemporal gait variables (26, 27), with a lack of consideration for unilateral abnormalities. Additionally, these methods are mostly based on fixed rhythms, lacking consideration for the heterogeneous symptoms of PD (28).

**Figure 1:**
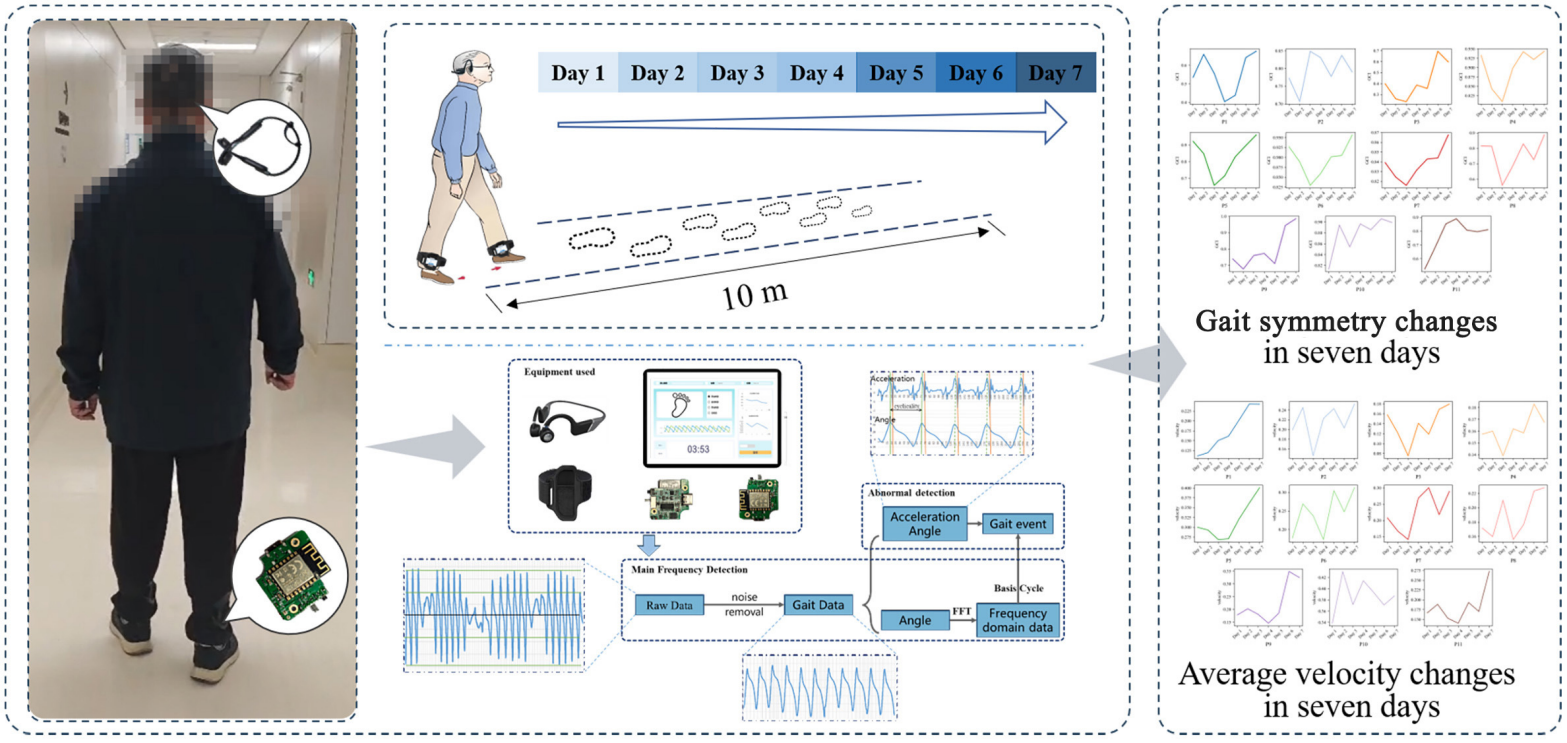
Personalized training method.

In the early-stage of PD, symptoms are typically unilateral, corresponding to asymmetric neuropathology of the basal ganglia (29, 30), meaning that the limbs are not affected symmetrically. This asymmetry significantly impacts patients' daily activities and reduces their quality of life, potentially leading to severe consequences such as falls and even death (31, 32). Meanwhile, existing studies have shown that treatment in the early-stages of PD can cover patients to improve motor function and quality of life (33–35). This study focuses on the gait characteristics of early-stage PD, then, proposes an intervention training method based on personalized auditory cues (as shown in Figure 1). We initially invited 15 patients with early-stage PD to undergo comparative training with and without auditory cues, and then invited 11 patients with early-stage PD to undergo a one-week rehabilitation program. Then explored the improvement of gait symmetry and gait variables by training under personalized auditory cues. The results indicate that after training accompanied by personalized auditory cues, the symmetry of spatiotemporal gait characteristics improved, along with improvements in spatiotemporal gait variables.

## 2 Methods

### 2.1 Participants

To assess the impact of personalized auditory cues on gait improvement, 15 patients were initially recruited to validate the effectiveness of the intervention, and then 11 patients were recruited for a week of training to explore the effect of continuous training. In this study, the patients were recruited from Beijing Friendship Hospital Affiliated to the Capital University of Medical Sciences. The inclusion criteria for patients were as follows: (1) clinical diagnosis of idiopathic PD based on the UK PD Society Brain Bank criteria (36); (2) Hoehn and Yahr (H&Y) scale ≤ 2.5; (3) presence of gait impairments; and (4) ability to walk without the assistance of walking aids. The exclusion criteria were: (1) atypical or secondary Parkinsonism; (2) presence of other diseases besides PD that affect gait or postural stability; (3) hearing impairment; and (4) dementia. PD patients with gait impairments were identified from their medical history and using the International Parkinson and Movement Disorder Society Unified Parkinson's Disease Rating Scale (MDS-UPDRS) part-II, and part-III (a score ≥1 on item 2.12 or 2.13 in MDS-UPDRS-II or a score ≥1 on item 3.10 or 3.11 in MDS-UPDRS-III). Table 1 presents the demographic and clinical information of the participants.

**Table 1:** Demographic characteristics of PD.

**Patient**	**Sex, M/F**	**Age (years)**	**H&Y**	**UPDRS I**	**UPDRS II**	**UPDRS III**
PD patients were trained for 1 day (*n* = 15)	7/8	72.23 ± 4.48	1.81 ± 0.32	10.41 ± 3.12	9.46 ± 2.96	23.84 ± 9.32
PD patients were trained for 7 days (*n* = 11)	4/7	73.5 ± 6	2.19 ± 0.25	10.12 ± 3.40	11.63 ± 3.78	27.38 ± 7.15

M, male; F, female; H&Y, Hoehn & Yahr; UPDRS I, unified Parkinson's disease rating scale part I; UPDRS II, unified Parkinson's disease rating scale part II; UPDRS III, unified Parkinson's disease rating scale part III.

The recruitment of subjects and experimental procedures for this study were approved by the ethics committees of Beijing Friendship Hospital, Capital Medical University (2024-P2-509-01). All participants provided written informed consent prior to participation and retained the right to withdraw from the study at any time without providing a reason. Their personal information was used exclusively for research purposes.

### 2.2 Personalized training method for PD patients

The heterogeneity of PD patients poses personalized requirements for training methods (28). To this end, we have proposed a personalized training method. Inertial Measurement Units (IMUs) are attached to the ankles of both the left and right feet to capture real-time gait data (as shown in Figure 1), and subsequently calculates the gait cycle for both the patient's left and right feet. Following this, based on the gait cycles of the left and right feet, a piece of music (auditory cues) that matches the patient's gait cycle is generated and played to the patient through bone-conduction headphones. Finally, the method incorporates a dynamic updating approach, which allows the music to be updated during the training process in response to the patient's actual performance.

#### 2.2.1 Neurological mechanisms of auditory cues to improve gait disorders in PD

Motor symptoms in PD patients are primarily caused by the loss of dopaminergic signaling in the basal ganglia region (37). Middle spiny neurons in the direct and indirect pathways of the basal ganglia circuit control motor facilitation and motor inhibition, respectively (38). PD patients have a dopaminergic deficit in the basal ganglia circuit, which in turn leads to motor symptoms. Motor rhythms are mediated by multiple regions of the brain that are not affected by PD, but damaged basal ganglia affect the function of these regions (39). Rhythmic stimulation by music stimulates the cerebellum to calibrate motor-sensory feedback signals for internal rhythms, forming a basal ganglia-sensory-motor cortex-complementary motor area circuit, which in turn compensates for the damaged basal ganglia (40).

Music, as an activation signal, can stimulate the motor system of PD patients to synchronize with musical rhythms through a rhythmic entrainment effect (41). Trained with musical cues, PD patients were able to activate the motor network associated with rhythm perception and regulate movement through music, resulting in improved gait (42). Imaging data suggest that musical cues increase functional neural connections between the auditory cortex and executive control networks and between executive control networks and the cerebellum (43). Although existing studies have demonstrated that the use of rhythmic auditory cues enhances the functional connection between auditory and motor areas and promotes the synchronization of walking rhythms with external music rhythms (44), thereby improving gait and inhibiting the progression of motor symptoms in PD patients. However, most of the existing studies are based on the use of fixed rhythm metronomes, and most of them focus on the improvement of bilateral spatial-temporal characteristics of gait and lack the consideration of unilateral onset in early PD patients.

#### 2.2.2 Personalized auditory cues generation

Gait performance varies significantly among different PD patients. Therefore, this method employs a spatial stepwise estimation approach to obtain gait information, including special gait patterns. IMUs are attached to the ankles of both the patient's left and right feet to acquire gait data. Subsequently, the frequency domain information of the gait data is utilized to determine the fundamental gait cycle of the PD patient, which serves as the basis for establishing the threshold of posture event intervals. Finally, based on the abrupt changes in ankle movement, combined with acceleration and angle data, gait events in the temporal sequence of gait data are identified. Considering the asymmetry of walking in PD patients, this study calculated the gait cycle of the patient's left and right feet respectively based on the imu data of the patient's left and right feet. The calculation of the gait cycle is as follows:


(1) $$\begin{array}{l} m=\underset{i\in \left[SP,\cdots \;,SP+D\right]}{\mathrm{argmax}}angle_{z}^{i} \end{array}$$

(2) $$\begin{array}{l} T=\underset{i\in \left[m,\cdots \;,m+\frac{D}{2}\right]}{\mathrm{argmax}}acc_{z}^{i}-\underset{j\in \left[m-\frac{D}{2},\cdots \;,m\right]}{\mathrm{argmax}}acc_{z}^{j} \end{array}$$

Wherein, *SP* is the starting point of sampling; $D=\frac{kf_{s}}{f_{k}}*\frac{1}{f_{s}}=\frac{k}{f_{k}}$ is the fundamental gait cycle, with *k* being the index of the corresponding point in the frequency domain, and *f*_*k*_ the frequency; *angle*_*z*_ represents the angular data along the z-axis; *acc*_*z*_ represents the acceleration data along the z-axis.

Based on the calculated gait cycles of the patient's left and right feet, the proposed method further calculates the number of gait cycles per minute for the patient. This number of gait cycles is defined as the target Beat Per Minute (BPM) of the auditory cue (music). The method searches the music database for music with a BPM that is closest to the target BPM, and then modulates the BPM to the target BPM to synchronize it with the patient's gait cycle, which is then used as the patient's auditory cue (music). A database of 60 pieces of music was created under the guidance of a rehabilitation physician. The music is rhythmically modulated to fit the gait cycle of all patients, and can be removed during training based on patient feedback to minimize the impact of the music itself.

#### 2.2.3 Auditory cue dynamic adjustment

During training, the gait performance of patients can also be influenced by various external environmental factors and their own physiological state. The personalized training method proposed in this paper considers these situations, using the patients' healthy side as a reference. Through auditory cues, the performance of the affected side is improved toward the level of the healthy side, enhancing limb symmetry and improving gait performance.

Based on the separate calculation of the gait cycles for each foot, the cycle results for the left and right feet are denoted as *TL* and *TR*, with the side having the longer cycle indicating the side with impaired motor function. The adjustment goal is: on the basis that the patient can follow the auditory cues (with a gait cycle and rhythm cycle difference of less than ±10%), the impaired side in early-stage PD patients is gradually adjusted until the cycles of the left and right feet become relatively consistent. To identify a dynamic adjustment scheme for the stimulation parameters, we conducted an extensive literature review, reviewed relevant studies on gait training (45, 46), auditory cues (47, 48), and rehabilitation of PD patients (49), invited three clinical experts (average years of practice 11.5 years) to participate in the design. And based on the literature review combined with the experts' professional knowledge and clinical practice, we initially determined the parameter ranges (cycle difference of 0%–30%, matching rate thresholds of 60%–100%, and iterative cycles of 30s–90s, stimulation parameter adjustment ratio 5%–10%). We then invited 3 patients with early PD for initial testing and further fine-tuned the parameters based on their feedback and gait improvement. From the experience of clinical experts as well as patient pre-tests: a iterative cycles of 40 seconds ensured that patients had sufficient time to adapt to the adjustment while allowing timely interventions based on their gait changes; a 60% matching rate threshold ensured that patients were able to follow auditory cues most of the time while avoiding being too stringent and preventing them from completing the training; a 5% stimulus parameter adjustment margin on the affected side yielded effective adjustments without inducing patient discomfort; and the 10% cycle difference allows patients to generate some motor errors. Based on the results of the literature review, clinical expertise, and preliminary testing, we set the iterative cycles to 40 s, the cycle difference to 10%, the matching rate threshold to 60%, and the adjustment of stimulation parameters on the affected side to 5%. This protocol was designed to ensure consistency with actual rehabilitation programs and patient needs, and to maximize the effectiveness and feasibility of the intervention.

For example, during patient training the method will use 40s as a judgment cycle, if the match between the patient's gait cycle and the BPM of the auditory cue (music) is greater than 60% during this cycle, the auditory cue will be generated in the next cycle according to the patient's side cycle growth rate of 5%. If the match between the patient's gait cycle and the BPM of the auditory cue is less than 60% in that cycle, the patient is considered unable to follow the current auditory cue, and the current auditory cue is kept unchanged to continue training. If the match between the patient's gait cycle and the BPM of the auditory cue is less than 60% for two consecutive cycles, the auditory cue will be generated according to the patient's side cycle deceleration of 5% during the next cycle until it returns to the initial level.

This dynamic adjustment can provide auditory cues that are synchronized with the patient's gait improvement, better adapting to the patient's gait performance changes, thus improving the training effect. At the same time, this dynamic adjustment can accelerate the improvement of the patient's lateral normal gait, shorten the training period, and improve the real-time and effectiveness of training.

### 2.3 Rehabilitation training procedure

(1) The day before first training, a clinician will conduct a secondary assessment of the patient using scales (MDS-UPDRS and H&Y scale).
(2) The training lasts for one week, with a daily requirement of 20 min of walking exercise (10 meters round trip) following auditory cues. The training process was designed with reference to existing clinical trials (50, 51), and the patient is not disturbed during the walking process in order to simulate as much as possible walking in a natural situation. Patients may terminate the exercise at any time if they experience discomfort and may also withdraw from the training at any time.
(3) All training sessions are conducted at Beijing Friendship Hospital, and it is ensured that the patients participate in the training while not taking any medication.
(4) At the start of the training, the patient is guided to stand at the starting position. After the experimenter gives the start command, the patient is instructed to begin walking naturally as they would in daily life, walking to the endpoint position (10 meters from the starting position), turning around, and walking back. During this time, when sound starts through the headphones, the patient is asked to try to step in time with the rhythm of the sound, continuously repeating this process until the training is completed.

### 2.4 Variables and analysis

Early-stage PD patients usually have motor symptoms in one limb, resulting in increased limb asymmetry. This asymmetry greatly affects the patient's life and can even result in falls and injuries. Therefore, this study focused on the improvement of gait symmetry by training methods. It also focuses on the effect of symmetry improvement on gait ability, which is reflected in the change of gait spatiotemporal variables. Spatiotemporal variables include parameters directly related to PD patients, such as velocity, stride length, turning time, swing phase, stance phase and double stance phase. These variables have been demonstrated to reflect gait performance in patients with PD (29, 30). For each participant, a total of 20 minutes of gait data for both the left and right foot was collected and the average spatiotemporal variables for that training session were calculated.

The improvement in gait performance is characterized by increased velocity, longer stride lengths, and reduced turning times. The improvement index (II) of velocity (left and right foot) and stride length (left and right foot) is calculated as follows:


(3) $$\begin{array}{l} \text{II}=\frac{P_{s}^{i}-P_{f}^{i}}{P_{f}^{i}} \end{array}$$

Where $P_{f}^{i}$ is the value of the i-th variable on the first day, $P_{s}^{i}$ is the value of the i-th variable on the 7th day.

The improvement index(II) of turning time is calculated as follows:


(4) $$\begin{array}{l} \text{II}=\frac{T_{f}-T_{s}}{T_{f}} \end{array}$$

Where *T*_*f*_ is the turning time on the first day, *T*_*s*_ is the turning time on the 7th day.

The improvement of spatiotemporal variables refers to the overall improvement in gait velocity, stride length, and turning time. The overall improvement index (OII) is calculated as follows:


(5) $$\begin{array}{l} \text{OII}=\frac{\sum_{i=1}^{n}II_{i}}{n} \end{array}$$

Where *n* is the number of spatiotemporal variables.

Gait symmetry is the consistency between the left and right foot of the patient during each training session in terms of gait velocity, stride length, swing phase, stance phase, and double stance phase, with the relative difference index (RDI) calculated as follows:


(6) $$\begin{array}{l} \text{RDI}=|\frac{L-R}{\frac{L+R}{2}}|\times 100\% \end{array}$$

Where *L* represents the variable value of the left foot, and *R* represents the variable value of the right foot.

Gait consistency index (GCI) is calculated as follows:


(7) $$\begin{array}{l} \text{GCI}=1-\frac{\sum_{i=1}^{n}|\frac{L_{i}-R_{i}}{\frac{L_{i}+R_{i}}{2}}|}{n} \end{array}$$

Where *n* is the number of spatiotemporal variables, *L*_*i*_ and *R*_*i*_ are the values of the ith variable for the left and right foot, respectively.

## 3 Results

Early-stage PD patients typically exhibit unilateral onset, leading to increased gait asymmetry. In this study, 15 PD patients were initially recruited to undergo one session of training with and without auditory cues, respectively, to compare the improvement effects of the proposed method on walking asymmetry. Then, 11 PD patients were recruited to perform a 1-week walking training with auditory cues to analyze the effects of multiple training sessions on the improvement of walking symmetry and spatiotemporal variables. We calculated the mean values of the spatiotemporal variables of the left and right feet at each time by using the imu data of the left and right feet during training. Then, we compared and analyzed the gait symmetry with the change of spatiotemporal variables.

### 3.1 Single training comparison

We verified the proposed method can improve walking asymmetry by comparing the walking symmetry with and without auditory cues. The results, as shown in Table 2, show that the symmetry of patients' walking with auditory cues is significantly better than without auditory cues (*t* = 4.9166, *p* = 0.0002), and the spatio-temporal variables of most of the patients are also significantly improved, such as Velocity of left (*Cohen*′*sd* = 0.736) and Turning time (*Cohen*′*sd* = −0.706), which indicates that the proposed intervention method is effective.

**Table 2:** Comparison of gait symmetry with and without auditory cues.

**Patient**	**Trainingconditions**		**Velocity**			**Stride length**			**Turning**	**Swing Phase**			**Stance phase**		
		**GCI**	**left/m/s**	**right/m/s**	**RDI**	**left/m**	**right/m**	**RDI**	**time/s**	**left**	**right**	**RDI**	**left**	**right**	**RDI**
P1	Non-intervention	92.788%	0.112	0.128	13.333%	0.160	0.169	5.471%	4.066	45.657%	43.178%	5.581%	54.343%	56.822%	4.460%
	Auditory cues	97.843%	0.13	0.135	3.774%	0.172	0.169	1.760%	3.783	43.434%	42.675%	1.763%	56.566%	57.325%	1.333%
P2	Non-intervention	83.323%	0.124	0.194	44.025%	0.160	0.179	11.209%	2.393	42.470%	45.296%	6.439%	57.530%	54.704%	5.036%
	Auditory cues	84.664%	0.143	0.196	31.268%	0.164	0.189	14.164%	2.317	41.953%	45.872%	8.925%	58.047%	54.128%	6.987%
P3	Non-intervention	75.615%	0.082	0.161	65.021%	0.134	0.165	20.736%	3.976	35.413%	38.153%	7.450%	64.587%	61.847%	4.335%
	Auditory cues	79.227%	0.087	0.15	53.165%	0.134	0.160	17.687%	2.785	35.633%	38.489%	7.704%	64.367%	61.511%	4.537%
P4	Non-intervention	89.694%	0.138	0.112	20.800%	0.160	0.149	7.120%	2.669	38.003%	34.921%	8.454%	61.997%	65.079%	4.852%
	Auditory cues	92.538%	0.139	0.124	11.407%	0.164	0.154	6.289%	2.756	38.328%	35.498%	7.667%	61.672%	64.502%	4.486%
P5	Non-intervention	88.809%	0.12	0.139	14.672%	0.155	0.175	12.121%	4.38	39.641%	44.014%	10.455%	60.359%	55.986%	7.517%
	Auditory cues	92.520%	0.123	0.135	9.302%	0.156	0.170	8.589%	3.72	40.476%	43.406%	6.985%	59.524%	56.594%	5.046%
P6	Non-intervention	54.661%	0.039	0.107	93.151%	0.118	0.154	26.471%	3.02	25.414%	38.880%	41.888%	74.586%	61.120%	19.846%
	Auditory cues	62.709%	0.046	0.106	78.947%	0.129	0.152	16.370%	2.66	26.082%	37.786%	36.650%	73.918%	62.214%	17.195%
P7	Non-intervention	79.592%	0.175	0.254	36.830%	0.197	0.243	20.909%	2.58	38.715%	44.521%	13.950%	61.285%	55.479%	9.944%
	Auditory cues	82.242%	0.18	0.241	28.979%	0.193	0.228	16.627%	2.586	37.923%	44.073%	15.001%	62.077%	55.927%	10.424%
P8	Non-intervention	85.887%	0.151	0.114	27.925%	0.176	0.161	8.902%	3.823	40.799%	45.614%	11.145%	59.201%	54.386%	8.479%
	Auditory cues	92.135%	0.148	0.123	18.450%	0.168	0.169	0.593%	3.278	41.597%	44.643%	7.063%	58.403%	55.357%	5.354%
P9	Non-intervention	90.378%	0.148	0.114	25.954%	0.150	0.141	6.186%	2.346	37.037%	38.529%	3.950%	62.963%	61.471%	2.399%
	Auditory cues	95.007%	0.146	0.127	13.919%	0.145	0.153	5.369%	2.248	37.613%	37.453%	0.426%	62.387%	62.547%	0.256%
P10	Non-intervention	77.008%	0.137	0.182	28.213%	0.094	0.135	35.847%	3.976	33.172%	39.633%	17.748%	66.828%	60.367%	10.159%
	Auditory cues	91.090%	0.163	0.174	6.528%	0.112	0.119	6.050%	3.148	33.462%	38.784%	14.732%	66.538%	61.216%	8.331%
P11	Non-intervention	75.895%	0.23	0.156	38.342%	0.185	0.112	49.158%	2.785	42.660%	40.493%	5.211%	57.340%	59.507%	3.708%
	Auditory cues	81.829%	0.234	0.168	32.836%	0.180	0.128	33.766%	3.481	40.520%	39.063%	3.663%	59.480%	60.938%	2.421%
P12	Non-intervention	68.579%	0.092	0.13	34.234%	0.138	0.250	57.732%	4.736	29.963%	37.500%	22.346%	70.037%	62.500%	11.374%
	Auditory cues	76.744%	0.097	0.128	27.556%	0.147	0.210	35.294%	3.273	30.769%	37.556%	19.866%	69.231%	62.444%	10.309%
P13	Non-intervention	73.508%	0.215	0.157	31.183%	0.206	0.132	43.787%	4.494	33.175%	40.382%	19.597%	66.825%	59.618%	11.400%
	Auditory cues	91.193%	0.205	0.174	16.359%	0.194	0.168	14.365%	4.181	35.027%	36.059%	2.903%	64.973%	63.941%	1.601%
P14	Non-intervention	69.515%	0.298	0.131	77.855%	0.244	0.194	22.831%	4.278	41.414%	36.364%	12.987%	58.586%	63.636%	8.264%
	Auditory cues	73.294%	0.309	0.139	75.893%	0.252	0.196	25.000%	4.293	37.507%	36.126%	3.749%	62.493%	63.874%	2.184%
P15	Non-intervention	79.373%	0.192	0.107	56.856%	0.100	0.086	14.615%	4.847	42.449%	45.165%	6.201%	57.551%	54.835%	4.834%
	Auditory cues	79.957%	0.208	0.125	49.850%	0.096	0.088	8.739%	4.106	40.393%	45.684%	12.294%	59.607%	54.316%	9.289%

Gait asymmetry improved in all patients with personalized auditory cues (*t* = 4.9166, *p* = 0.0002), Cohen's d reached 1.2695, in with the maximum improvement rate reaching 17.685% and the minimum improvement rate at 0.584%. Additionally, the personalized auditory cues significantly improved difference in left- and right-footed gait velocity (*t* = 8.055, *p* = 0.000001, *Cohen*′*sd* = 2.080) and difference in stride length (*t* = 3.199, *p* = 0.006, *Cohen*′*sd* = 0.830). This result shows that auditory cues are able to enhance patients' gait symmetry, especially with personalized auditory cues to guide their gait performance. Personalized auditory cues were able to provide rhythms that matched the patient's gait performance according to his or her gait characteristics, thus helping the patient to better coordinate his or her left- and right-side gait and reduce asymmetry. This improvement was not only reflected in the data, but also in the patients' subjective perceptions, which indicated that they were more stable and confident when walking with auditory cues.

Further, the improved symmetry also had an effect on the gait spatiotemporal variables. Most of the patients' patient-side spatiotemporal variable improved, and although some of the patients' normal-side variables were affected, the combined left and right variables showed an overall trend of improvement in spatiotemporal variables. This result shows that auditory cues can improve patients' gait spatiotemporal variables, and that personalized music can activate the motor network associated with rhythm perception, modulating movement through music and thus improving gait performance. This improvement may be related to the activation of the brain motor cortex by auditory cues, especially the modulatory effect on the basal ganglia-thalamo-cortical loop, which helps patients to better control their gait. In addition, we found that most of the patients showed a decrease in turning time in response to auditory cues, which not only indicates an improvement in gait control, but also reflects the patients' performance enhancement in complex motor tasks.

Individually, the optimal improvement was achieved with *P*13, in which we found that although the spatiotemporal parameters improved on both the symmetry and patient sides of the patient, they decreased on the normal-side. But when combining the parameters on both sides, the spatiotemporal parameters showed an overall trend of improvement, and the patients reported that they walked more steadily in this situation. This phenomenon may indicate that auditory cues may temporarily interfere somewhat with the normal-side while improving function on the patient side, but this interference does not affect the overall gait improvement. There were also a few patients for whom the improvement was not very significant, such as *P*15(0.584%) and *P*2(1.341%), but in terms of spatiotemporal variables these patients also showed an improved effect of training with auditory cues. Individual performance also further validates the effectiveness of the proposed method.

Overall, the proposed method demonstrates effectiveness in improving gait disorders in patients with early-stage PD. The method is able to give real-time prompts and guidance based on the patient's problems during walking, which in turn improves gait performance. This guidance is what is lacking during free walking, as patients often do not realize they have a problem with their gait, or if they know they have a problem with their gait, they do not know how to correct it. With personalized auditory cues, patients can receive real-time feedback during walking to better adjust their gait.

### 3.2 Analysis of gait performance after 7 days of training

Further to verify the effect of the proposed method after multiple training sessions, we invited 11 patients with early-stage PD to undergo 7 days of training and then analyzed the changes in gait symmetry and spatiotemporal variables. The gait symmetry and spatiotemporal variables of all patients improved after 7 days of training, indicating that the proposed method can improve the gait performance of early-stage PD patients.

#### 3.2.1 Analysis of gait symmetry

The changes in gait symmetry of each patient after training are shown in Table 3. Overall, gait symmetry significantly improved in each patient after continuous training (*t* = 4.391, *p* = 0.001, *Cohen*′*sd* = 1.320), with an average improvement rate of 11.803%. Of these, the personalized auditory cues significantly improved difference in left- and right-footed gait velocity (*t* = 3.596, *p* = 0.005, *Cohen*′*sd* = 1.080) and difference in stride length (*t* = 2.412, *p* = 0.037, *Cohen*′*sd* = 0.730). Among them, *P*26 showed the best improvement with an improvement rate of 25.367%, and *P*2 showed the worst improvement with an improvement rate of 1.787%. On an individual basis, patients with poor initial symmetry improved more significantly (*P*16, *P*19, *P*23, *P*24, *P*25, *andP*26), which suggests that the individualized training method for the characteristics of unilateral onset of early-stage PD patients is effective. In detail, after continuous training, the *RDI* of each spatiotemporal variable of each patient showed a decreasing trend, and from Table 4, the decrease of *RDI* did not lead to the decrease of spatiotemporal variables, on the contrary, most of the spatiotemporal variables were significantly improved. This proves that the proposed method can effectively improve walking symmetry.

**Table 3:** Comparison of gait symmetry between day 1 vs. day 7.

**Patient**	**Time**	**GCI**	**Improvement**	**RDI of**	**RDI of**	**RDI of**	**RDI of**	**RDI of Double**
			**of GCI**	**Velocity**	**Stride length**	**Swing phase**	**Stance phase**	**Stance phase**
P16	Day 1	54.685%	19.188%	78.027%	42.164%	12.185%	12.216%	81.99%
	Day 7	73.873%		31.753%	35.019%	32.022%	27.223%	4.62%
P17	Day 1	76.986%	1.787%	47.236%	39.515%	9.544%	5.461%	13.31%
	Day 7	78.773%		42.376%	49.669%	6.589%	3.770%	3.73%
P18	Day 1	60.238%	20.889%	20.253%	89.052%	16.028%	11.976%	61.50%
	Day 7	81.126%		10.585%	33.809%	0.506%	0.435%	49.03%
P19	Day 1	89.746%	4.454%	13.333%	5.846%	7.193%	4.113%	20.78%
	Day 7	94.200%		0.601%	9.985%	6.053%	3.505%	8.86%
P20	Day 1	92.006%	3.934%	13.333%	9.311%	4.881%	2.665%	9.78%
	Day 7	95.940%		5.743%	8.209%	2.857%	1.533%	1.96%
P21	Day 1	92.370%	4.745%	10.169%	15.144%	1.816%	0.878%	10.14%
	Day 7	97.114%		4.160%	2.763%	1.661%	0.899%	4.94%
P22	Day 1	84.606%	4.172%	35.577%	15.296%	7.311%	4.910%	13.88%
	Day 7	88.778%		23.143%	20.169%	1.142%	0.727%	10.93%
P23	Day 1	82.132%	9.730%	22.741%	36.158%	10.771%	5.986%	13.68%
	Day 7	91.862%		14.035%	16.208%	4.202%	2.364%	3.88%
P24	Day 1	74.834%	23.850%	46.067%	48.654%	0.814%	0.459%	29.83%
	Day 7	98.684%		0.615%	2.051%	0.757%	0.439%	2.72%
P25	Day 1	81.348%	11.718%	32.792%	33.471%	0.787%	0.480%	25.73%
	Day 7	93.065%		15.504%	15.059%	1.169%	0.712%	2.23%
P26	Day 1	55.440%	25.367%	102.703%	104.754%	8.099%	3.394%	3.85%
	Day 7	80.807%		45.138%	38.890%	7.227%	3.640%	1.07%

**Table 4:** Comparison of improvements in gait variables.

**Patient**	**OII**	**Improvement of**	**Improvement of**	**Improvement of**	**Improvement of**	**Improvement of**	**Improvement of**
		**Velocity (left)**	**Velocity (right)**	**Stride length (left)**	**Stride length (right)**	**Turning time**	**GCI**
**P16**	131.550%	81.290%	200.000%	203.513%	226.897%	58.412%	19.188%
**P17**	25.419%	24.390%	30.921%	27.238%	14.340%	53.835%	1.787%
**P18**	3.937%	–2.299%	33.099%	–47.606%	–2.975%	22.517%	20.889%
**P19**	12.889%	12.925%	–0.595%	1.679%	5.983%	52.890%	4.454%
**P20**	24.804%	28.750%	38.929%	35.875%	37.383%	3.953%	3.934%
**P21**	53.748%	71.505%	82.143%	83.841%	108.132%	–27.879%	4.745%
**P22**	29.096%	31.837%	49.708%	31.837%	25.518%	31.508%	4.172%
**P23**	27.421%	39.474%	27.749%	48.498%	21.197%	17.880%	9.730%
**P24**	70.420%	136.496%	48.858%	149.635%	48.858%	14.823%	23.850%
**P25**	9.468%	5.838%	26.148%	9.398%	31.896%	-28.188%	11.718%
**P26**	28.664%	32.540%	160.494%	–36.402%	37.236%	–47.250%	25.367%

**Figure 2:**
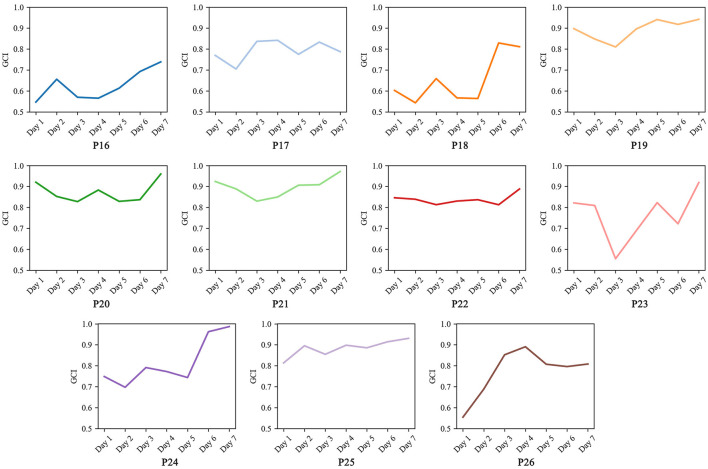
Gait symmetry changes in 7 days.

For further analysis of the change in gait symmetry during training, we calculated the change in symmetry over the seven training sessions for each patient separately, as shown in Figure 2. The trends were different and varied greatly from patient to patient, which may be due to a combination of PD heterogeneity, individual differences, and state differences, a condition that partly validates the need for a personalized therapy for PD patients. But, most patients exhibited a pattern of initially decreasing followed by an increase, which may be due to the initial inability to adapt to the rhythm of the music, with external auditory cues disrupting the patient's original walking rhythm. Each patient's symmetry changes showed fluctuations; as indicated in Table 3, these may be attributed to temporary asymmetries caused by the improvement of spatiotemporal variables. Overall, the symmetry of each patient tends to show a gradual improvement trend. This is also able to show that the proposed method is able to improve the asymmetry caused by unilateral onset in patients with early-stage PD and improve the abnormal gait.

#### 3.2.2 Spatiotemporal variable improvements

To validate the efficacy of the proposed method in improving abnormal gait in PD patients, we calculated the improvement indices of spatiotemporal variables, as presented in Table 4. Overall, the gait performance of each patient improved with a mean *OII* of 37.947%. There were significant improvements in left-foot velocity (*t* = 4.613, *p* = 0.001), right-foot velocity (*t* = 6.250, *p* = 0.0001), and right-foot stride length (*t* = 4.004, *p* = 0.0025), as well as large effect sizes for left-foot velocity (*Cohen*′*sd* = 1.391), right-foot velocity (*Cohen*′*sd* = 1.884), and right-foot stride length (*Cohen*′*sd* = 1.201), and moderate effect sizes for turning time (*Cohen*′*sd* = 0.539). This result shows the generalizability of the proposed method in optimizing gait in patients with early-stage PD. However, there were significant differences in the improvement results among different patients, which were closely related to their initial states, individual abilities, and adaptations to the intervention methods.

Among them, *P*16 showed the most significant improvement, with *OII* reaching 131.550%. This significant improvement may be related to the poor initial state of this patient. Patients with poorer initial status typically exhibit more room for improvement, and thus their gait parameters improved more significantly after receiving the intervention. In contrast, *P*18 showed a weaker improvement with *OII* of only 3.937%. This may be due to the fact that this patient's initial state was better with relatively normal gait function, thus the room for improvement was limited. Nevertheless, the gait of *P*18 remained stable, which also reflects the robustness of the proposed method. In terms of the combined improvement in each spatiotemporal variable, the proposed method can effectively optimize the gait performance of PD patients. All patients showed an improvement trend in spatiotemporal variables, such as parameters of stride length and velocity to different degrees. However, it is worth noting that certain gait variables did not improve or even showed a slight deterioration in some patients. This phenomenon may be related to the patient's own ability level, physical condition, and adaptation to the intervention method. In addition, we observed interactions between gait variables. For example, in *P*26, an increase in right-footed gait may lead to a decrease in left-footed gait; in *P*18, an increase in speed may lead to a decrease in gait. This mutual constraint between variables may be due to the patient's need to maintain balance and stability during walking. However, from the point of view of walking symmetry, this reduction may instead lead to a more balanced gait variable between the right and left foot, thus improving the overall coordination of the gait. Thus, although some variables may not have improved as expected, the change is still positive from the perspective of overall gait optimization.

**Figure 3:**
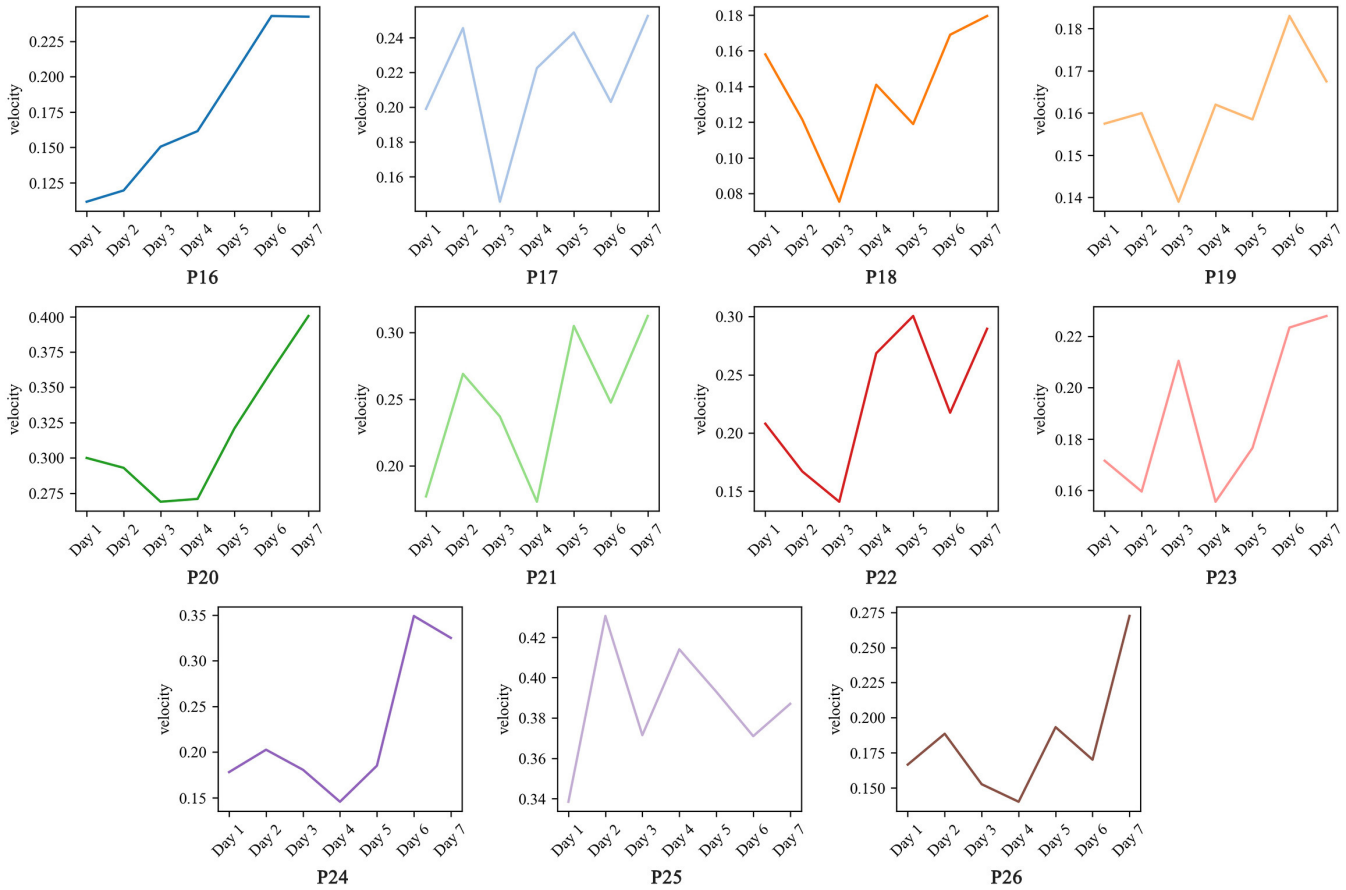
Average velocity changes in 7 days.

To visualize the changes in the spatiotemporal variables during training, we selected the velocity, which is generally of greater interest in current research, and calculated the average velocity of the left and right feet for the 7-day visualization, as shown in Figure 3. As can be seen from the figure, the velocity of most patients showed a fluctuating upward trend during the training process. This fluctuation may be due to the change in tempo of the music and the need for patients to constantly adapt to the new tempo, resulting in fluctuations in velocity in the short term. However, in the long term, all patients adapted to the proposed method within 1–2 days and their speed increased significantly after adaptation. In addition, the great variability in patients' speed changes further validates the need for individualized treatment of PD patients. There are significant differences in the responses of different patients to the intervention methods, so in practice, the intervention strategies need to be adapted to the specific conditions of the patients in order to achieve the best therapeutic effect.

## 4 Discussion

In this study, an intervention training method based on personalized auditory cues was proposed for the intervention of motor symptoms in patients with early PD. The method generates personalized music (auditory cues) based on the patient's gait data and can be dynamically adjusted according to the patient's actual gait performance. By analyzing the patients' gait data, we found that symmetry of patients' walking with auditory cues is significantly better than without auditory cues (*t* = 4.9166, *p* = 0.0002). Gait symmetry improved in each patient after 7-day training (*t* = 4.391, *p* = 0.001, *Cohen*′*sd* = 1.320), there were significant improvements in left-foot velocity (*t* = 4.613, *p* = 0.001), right-foot velocity (*t* = 6.250, *p* = 0.0001), and right-foot stride length (*t* = 4.004, *p* = 0.0025). This work aims to promote relief of motor symptoms in PD, help patients better manage the disease, and ultimately enable in-home self-management of PD through more advanced computerized methods and human-computer interaction technologies. Several limitations of this study should be acknowledged. The number of subjects was relatively small, which limits the generalizability of the results. But early-stage PD patients are difficult to recruit, and often they are diagnosed in the mid to late stages when symptoms are more severe. And, there was no follow-up period, and changes in patients' gait performance after cessation of training are not clear. There are a number of insights that could help researchers in the field of PD disease or motor symptom intervention to benefit from this work.

### 4.1 Early stages of PD: a critical period for intervention training

Rehabilitation training provides an avenue for the alleviation of motor symptoms in PD (12, 13), but most of the existing auditory cue-based training methods target bilateral symptoms in mid- to late-stage PD, e.g., freezing of gait (22, 25), focusing on the improvement of bilateral spatiotemporal parameters (26, 27). In addition, auditory cues mostly use fixed rhythms (21, 24) and lack adaptation for patient improvement during training. However, interventions in the early stages are more meaningful in slowing down the progression of the disease (52). We observed during training that early-stage PD patients are often unaware of their gait issues or, if aware, may not fully understand the nature of the problem. Auditory cues can activate the motor cortex, aiding patients in controlling their gait performance, particularly when personalized auditory cues are designed to guide their walking patterns. Personalized auditory cues can help patients better adjust their gait by providing rhythms that match their gait performance. Meanwhile, the results of the experiment show that the proposed training method can indeed improve patients' gait performance, which has a positive effect on the management of PD. The proposed method complements existing methods by providing a training approach that starts early in the disease to slow down the progression of motor symptoms.

### 4.2 Heterogeneity and individual differences: the significance of individualization

Although the onset of PD is generally characterized by a unilateral onset, the subsequent motor symptoms show significant heterogeneity (37, 38). This heterogeneity is reflected in the fact that the type, severity, and rate of progression of symptoms vary from person to person (29, 37). Therefore, an individualized treatment plan is particularly important in clinical rehabilitation. A professional rehabilitator is usually involved to conduct a comprehensive assessment and to develop a targeted training program accordingly (12, 13). However, the reality is that there are a number of challenges. On the one hand, the rehabilitator is unable to accompany the patient at all times and monitor his/her condition and rehabilitation progress in real time, which leads to a certain lag in the adjustment of rehabilitation strategies and an inability to respond to the patient's changing needs in a timely manner. On the other hand, even when receiving the same rehabilitation training, there are significant differences in the responses of different patients. From the results of the seven-day training in this paper, the performance of all patients is different, and although the overall trend of fluctuation is upward, this fluctuation demonstrates obvious individual variability. This variability is rooted in the heterogeneity of the disease itself, the patient's own physical condition, adherence to rehabilitation training, psychological state, and a variety of other factors that can affect gait improvement. Therefore, individualization is especially critical in PD rehabilitation.

### 4.3 Encouragement and self-confidence: the psychological role of training

“When I was diagnosed, I strongly urged my doctor to give me some medication, even though I wasn't symptomatic yet, because I wanted to do something to deal with the disease.” This positive attitude is crucial in people with Parkinson's disease. However, more commonly, people with PD may unconsciously give themselves negative mental cues after learning about their disease, such as, “I have PD, I have to be extra careful when I walk, and I have to watch my step at all times.” Through further conversations with clinicians, we realized that this phenomenon is very common among PD patients. After diagnosis, many patients experienced non-pathological aggravation of motor symptoms due to fear and anxiety of the disease, as well as excessive psychological cues, creating a vicious cycle of “the more afraid, the worse” (53). This psychological factor has a significant negative impact on the patient's motor function and may even aggravate the disease (54).

The proposed training method, in addition to physical training for motor function, also encourages patients on a psychological level. Participants reported that they felt more confident and empowered to walk to the rhythm. This positive psychological change may be due to a sense of accomplishment from the training, a renewed sense of awareness of one's abilities, and a sense of control from actively participating in the rehabilitation process. When patients see that they are able to improve their gait through training, they become more confident in their abilities, which leads to less negative self-references and more self-confidence. This positive psychological state, in turn, will promote the improvement of motor function, forming a virtuous cycle of “the more you train, the better you get.” Therefore, in the rehabilitation training of PD, we cannot ignore the importance of psychological factors.

### 4.4 In-home training: needed for long-term chronic diseases

Long-term management of PD, a chronic, progressively developing neurodegenerative disorder, is a process that occurs throughout the patient's life course (52). Due to the long-term nature of the disease, most of the management actually takes place in the home environment; therefore, home training is crucial for PD patients (55). It not only helps patients maintain or improve motor function, but also improves quality of life and delays disease progression. Although all of the training in this article was performed at Beijing Friendship Hospital of Capital Medical University, patients are fully capable of completing the training independently and safely in their home environment without too much involvement of a physician, after they understand the basic principles of the training and the correct way to use it under the guidance of a healthcare professional. The advantage of this home training mode is that it breaks the dependence of traditional rehabilitation training on venues and equipment, enabling patients to carry out rehabilitation exercises anytime and anywhere. The proposed method provides a practical pathway for home training in early-stage Parkinson's disease patients and fulfills the urgent need for patient intervention in the early stage of the disease, when patients are not yet heavily dependent on medication, and is expected to become an important part of the long-term management strategy for PD.

## Data Availability

The datasets presented in this article are not readily available because gait data from patients with early Parkinson's disease were used only for this institutional study. Requests to access the datasets should be directed to Xinjin Li, lixinjin2022@iscas.ac.cn.
